# Bisphosphonates therapy for osteoarthritis: a meta-analysis of randomized controlled trials

**DOI:** 10.1186/s40064-016-3359-y

**Published:** 2016-10-03

**Authors:** R. L. Xing, L. R. Zhao, P. M. Wang

**Affiliations:** Affiliated Hospital of Nanjing University of Chinese Medicine, Hanzhong Road 155#, Nanjing, Jiangsu Province China

**Keywords:** Bisphosphonates, Osteoarthritis, Meta-analysis

## Abstract

High-turnover type bone metabolism derangement has been considered to be one of the major causes of osteoarthritis (OA). Bisphosphonates can attach to hydroxyapatite binding sites on bony surfaces, particularly those which are undergoing active bone resorption. To evaluate the effectiveness of bisphosphonates in OA treatment, literature databases were searched from inception to February 28, 2016 for clinical studies of bisphosphonates for OA treatment. All randomized controlled trials in which bisphosphonates therapy was compared with a placebo or a conventional medication, were selected. 15/1145 studies were eligible for analysis, which included 3566 participants. Bisphosphonates therapy improved pain, stiffness and function significantly in OA assessed by the Western Ontario and McMaster Universities Arthritis Index scale (MD = 4.59; 95 % CI 2.83–6.34; P < 0.00001; MD = 1.43; 95 % CI 0.83–2.23; P = 0.0005; MD = 2.01; 95 % CI 1.27–2.75; P < 0.00001). Bisphosphonates also reduced osteophyte score significantly (MD = −0.51; 95 % CI −0.84 to −0.19; P = 0.002). However, no significant differences were found in subjective improvement, osteoarthritis progression, the number of required acetaminophen treatment or joint replacement. In conclusion, bisphosphonates therapy is effective in relieving pain,stiffness and accelerating functional recovery in OA. Limitations of the studies we analysed included the differences in duration of bisphosphonates use, the doses and types of bisphosphonates and the lack of long-term data on OA joint structure modification after bisphosphonates therapy. More targeted studies are required to evaluate on the effectiveness of bisphosphonates for OA treatment.

## Background

Osteoarthritis (OA) is the most common form of arthritis. It is a major cause of disability among the ageing population. The affected joint is undergoing a complex combination of degradative and reparative processes (Collins et al. 2016a; Palazzo et al. 2016), the mechanism of which is still unclear. There are currently no treatments that delay or halt OA progression.

The main clinical manifestations include pain, swelling and disability caused by topical cartilage loss, subchondral bony changes, osteophyte formation and synovitis (Liu et al. 2016). OA has long been believed as a cartilage disease, but more recent evidence suggests that periarticular bone abnormality is also involved in the disease initiation and progression (Kalunian 2016). Decreased bone mineral content and trabecular numbers in subchondral bone structure in the early OA have been observed by magnetic resonance imaging (Madry et al. 2016). High-turnover type bone metabolism derangement has been considered as a main cause of OA (Collins et al. 2016b).

Previous experimental studies on bone anti-resorptive agents for OA have shown promising results (Fenty et al. 2012). The Duncan-Hartley guinea pig model is a widely used spontaneous model of OA progression, which is characterized by subchondral bony changes (Sun et al. 2015). In rat anterior cruciate ligament transection (ACLT) models of KOA, alendronate could protect cartilage from degeneration and inhibit subchondral bone remodeling (Strassle et al. 2010).

Bisphosphonates can inhibit bone resorption and therefore they are the mainstream medications for osteoporosis. But for OA treatment, there is no official statement or guideline for bisphosphonates therapy. Recently, more and more evidence have shown bisphosphonates are effective in OA treatment. Bisphosphonates can suppress local bone turnover or inhibit the level of local pro-inflammatory cytokines. Further studies confirmed that defective subchondral bone metabolism in OA could alter chondrocytes during subchondral bone remodeling. Increased subchondral bone turnover may contribute to pain in OA, which may be relieved by targeting osteoclasts. Although bisphosphonates therapy seems to have positive effects on OA, these effects have not been extensively studied. To our knowledge, only one systematic review and meta-analysis was conducted with a limited number of poor-quality RCTs, which demonstrated limited evidence of bisphosphonates for pain relief in OA (Davis et al. 2013). Moreover, osteoarthritis progression, required acetaminophen treatment and joint replacement were not analyzed.

Therefore, this review aims to summarize the results of these clinical trials and evaluate the clinical effects, which may be useful to clinical practice. This meta-analysis was conducted in accordance with Cochrane guidelines (Higgins and Green 2011).

## Methods

### Search strategies

All searches were conducted from database MEDLINE, PubMed, EMBASE, and the Cochrane Central Register of Controlled Trials (CENTRAL) from inception to February 28, 2016. The MeSH terms we used were (Osteoarthritis) AND (OA) AND (Bisphosphonates). To ensure a more complete meta-analysis, we used a maximally sensitive search for RCTs according to the Cochrane Highly Sensitive Search Strategy. Systematic review and meta-analysis were manually searched as references for included studies.

### Inclusion criteria

The inclusion criteria were (1) RCTs comparing bisphosphonates with any control methods, include a placebo or a conventional medication. And published as peer-reviewed indexed papers; (2) patients with established OA administering medication or other control interventions; (3) studies detailing the type and dosage of medications and treatment course; (4) primary outcomes included visual analogue scale (VAS) pain score, WOMAC pain, stiffness, function score and osteophyte score, while secondary outcomes included subjective improvement, osteoarthritis progression and the number of required acetaminophen treatment or joint replacement. Studies reported at least two of the primary outcomes. (5) Literature in English.

### Exclusion criteria

The exclusion criteria were (1) non-randomized controlled trials; (2) studies without available data; (3) duplicate publications among authors or centers.

### Data extraction and quality assessment

Data from all eligible papers were extracted and independently verified. Data extracted included study characteristics, patient characteristics, primary and secondary outcomes.

Study characteristics included study design, name of first author, sample size and follow-up period. Patient characteristics included the number of patients, gender, affected joint, type of intervention and dosage. If a study reported the outcomes of multiple doses of bisphosphonates, only data of the maximum dose were extracted for analysis. If a study reported the outcomes of multiple time points after treatment, only data of the final follow-up time point was extracted for analysis.

The methodological quality of each included study was evaluated in accordance with the Cochrane Collaboration’s risk of bias tool (Higgins and Green 2011), which used the following items as random sequence generation, allocation concealment, blinding of outcome assessors,patients and other participants, incomplete outcome data, selective reporting of outcomes and other biases. The bias risk of each item was graded as low, high or unclear.

### Statistical analysis

All statistical analyses were conducted with RevMan version 5.3 software (provided by Cochrane Collaboration). Heterogeneity was evaluated with Q tests and *I*^2^, and P < 0.10 was determined as significant. If there was no or low heterogeneity then the fixed-effects model was used. Otherwise, the random-effects model was used. The risk ratio (RR) was calculated for dichotomous data, and weighted mean differences (WMD) or standard mean differences (SMD) were used for continuous variables. Both differences were presented with 95 % CI. For continuous variables, if data were presented with medians and ranges, then the means and SDs were calculated according to Hozo et al. (2005). If the study presented the median and interquartile range, the median was treated as the mean, and the interquartile ranges were calculated using 1.35 SDs, as described in the Cochrane handbook.

## Results

### Search results

**Fig. 1:**
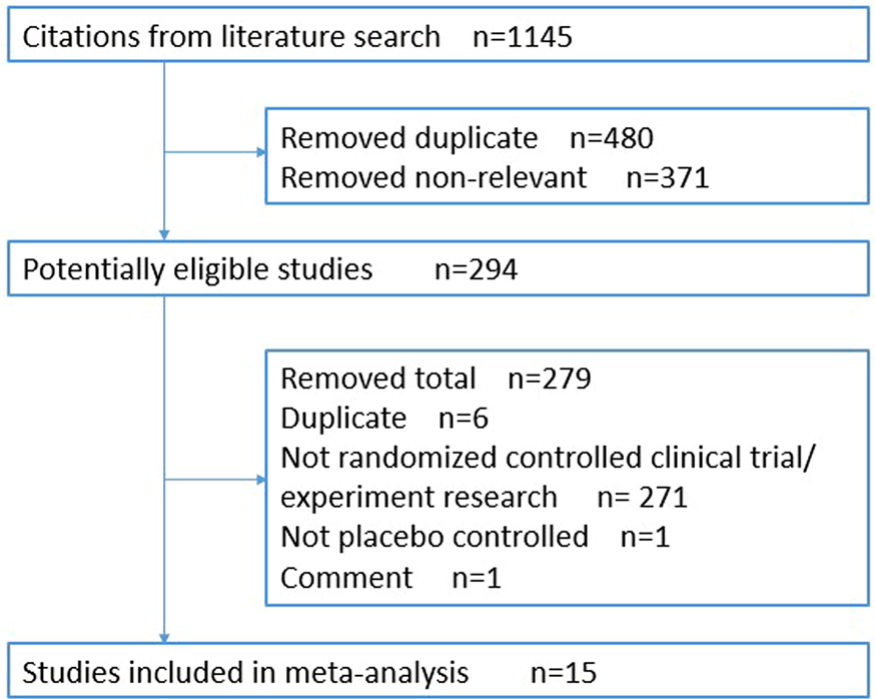
Flow chart of study selection

The results of the literature search strategy identified a total of 1145 papers. 294 full texts were reviewed and a total of 15 papers were deemed eligible and included in this systematic review (covering a total of 3566 patients, including 1517 in bisphosphonates group and 2049 in control groups) (Fig. 1). (Neogi et al. 2008; Rossini et al. 2015; Laslett et al. 2014; Nishii et al. 2013; Arti and Azemi 2012; Laslett et al. 2012; Saviola et al. 2012; Fujita et al. 2011; Rossini et al. 2009; Fujita et al. 2009; Bingham et al. 2006; Spector et al. 2005; Carbone et al. 2004; Fujita et al. 2001).

### Characteristics of included studies

Of the 15 eligible studies, 1517 patients received bisphosphonates therapy, and 2049 patients received control interventions. The baseline characteristics were similar among these trials (Table 1).

**Table 1:** Characteristics of 15 RCTs included

Authors	Study design	Number of patients	Age (year)	Affected joint	Diagnostic criteria	Duration (month)	Outcome meature	Drugs
Neogi et al. (2008)	Randomised case–control	200	65.8 ± 6.1 66.1 ± 6.4	Spine	Radiographs	24	OST, DSN	Alendronate VS placebo
Rossini et al. (2015)	Double-blind randomized	80	66 ± 6	Knee	Radiographs	3	WOMAC, VAS	Clodronate VS placebo
Laslett et al. (2014)	Observational cohort study	323	59.8 ± 8 66.7 ± 7.4	Knee	Radiographs	60	VAS, WOMAC, JSW	Bisphosphonates used VS Non-users
Nishii et al. (2013)	Non-blinded randomised	42	54.7 ± 8.5 58.3 ± 8.8	Hip	Radiographs	24	WOMAC, VAS, JSW	Alendronate VS calcium lactate
Arti and Azemi (2012)	Double-blind randomized	130	60.9 ± 9.9	Knee	Radiographs	3	WOMAC	Alendronate VS glucosamine
Laslett et al. (2012)	Double-blind randomised	53	64.2 ± 8.2 60.4 ± 7.3	Knee	Radiographs	12	VAS,KOOS;	Zoledronic VS placebo
Saviola et al. (2012)	Non-randomised case control	29	60.0 ± 7.1 63.5 ± 7.4	Hand	Radiographs	24	VAS, HS, NOSPJ	Clodronate VS HCQ
Fujita et al. (2011)	Randomised case–control	38	69 ± 8 68 ± 9	Spine, knee	Radiographs	6	VAS, SF-36	Risedronate VS Elcatonin
Rossini et al. (2009)	Partially blinded randomised	150	64.7 ± 7.4 65.2 ± 6.9	Knee	Radiographs	18	VAS, LI	Clodronate VS Hyaluronic acid
Fujita et al. (2009)	Non-randomised case–control	100	68 ± 9.0 66 ± 8.0	Spine, knee	Radiographs	7	VAS	Alendronate VS Calcium
Buckland-Wright (2007)	Double-blind randomized	627	60.3 ± 2.6 63.1 ± 2.3	Knee	Radiographs	24	JSW	Risedronate VS placebo
Bingham et al. (2006)	Double-blind randomized	2483	60.7 ± 0.5 60.2 ± 0.5	Knee	Radiographs	24	WOMAC, JSW,	Risedronate VS placebo
Spector et al. (2005)	Double-blind randomised	285	63.8 ± 0.9 63.2 ± 0.8	Knee	Radiographs	12	WOMAC; PGA	Risedronate VS placebo
Carbone et al. (2004)	Cross sectional cohort study	818	74.8 ± 2.9 74.8 ± 2.9	Knee	Radiographs	36	Modified WOMAC	Alendronate VS placebo
Fujita et al. (2001)	Non-blinded randomised,	80	65 ± 7	Spine, knee	Radiographs	12	VAS	Etidronate VAS placebo

*OST* spinal osteophytes, *DSN* disc-space narrowing, *WOMAC* Western Ontario McMaster Universities (WOMAC) Osteoarthritis Index, *VAS* Visual Analogue Scale, *JSW*: joint space width, *HS* hand strength, *NOSPJ* number of swollen and painful joints, *PGA* Patient Global Assessment, *LI* Lequesne Index, *KOOS* Knee Injury and OA Outcome Score

### Methodological quality of studies

**Fig. 2:**
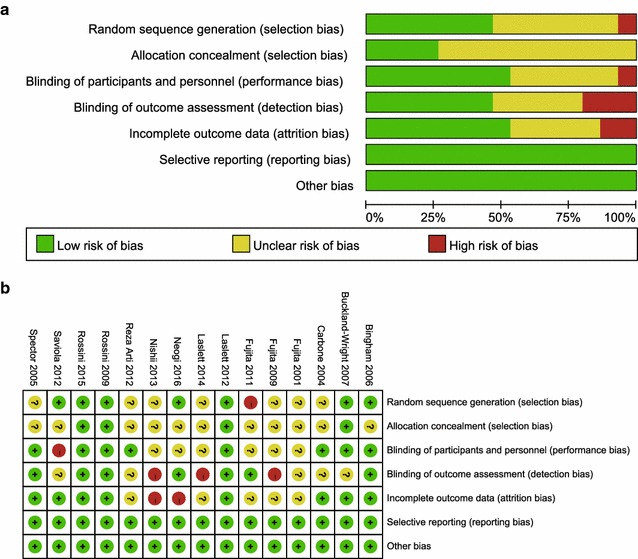
Methodological quality assessment according to the Cochrane Collaboration’s Risk of Bias tool study

The quality assessment of the trials was performed using the Cochrane Collaboration’s Risk of Bias tool. The results of quality assessment illustrated there were some methodological limitations in these studies, quality of which was moderate (Fig. 2). Random sequence generation was described clearly in 6 studies (40 %) and unclearly in 8 studies (53 %). 3 studies (20 %) had a high risk of bias relating to allocation concealment, and 12 studies (80 %) had an unclear risk of bias relating to allocation concealment. Only 7 studies (46.7 %) blinded outcome assessors and the blindness in 5 studies (33.3 %) were unclear. All RCTs had a low risk of bias relating to incomplete outcome data and selective reporting. Biases relating to other aspects were unclear.

## Meta-analysis

### Primary outcome parameters

**Fig. 3:**
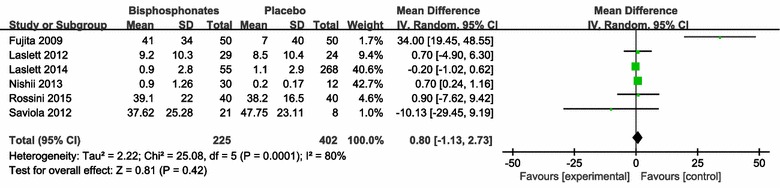
Change of Visual Analogue Scale (VAS) Pain Score

VAS pain score: the results of 6 studies (627 patients) with combined data indicated that bisphosphonates therapy improved pain nosignificantly assessed by VAS pain scores (MD = 0.80; 95 % CI −1.13 to 2.73; P = 0.42) compared with respective control group (Fig. 3).

**Fig. 4:**
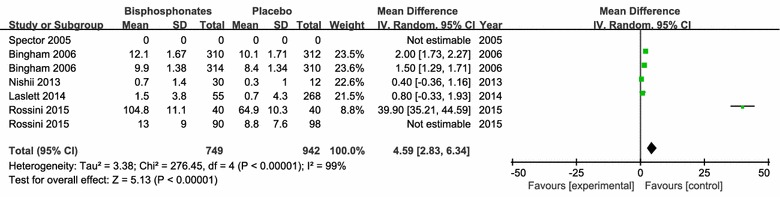
Change of WOMAC-Pain. The first Bingham et al. (2006) refers to the outcomes from 44 European centers (11 countries), the second Bingham refers to the outcomes from 42 centers in North America (US and Canada); The first Rossini et al. (2015) refers to the outcomes of 8 weeks follow-up, the second Rossini et al. (2015) refers to the outcomes of 16 weeks follow-up

WOMAC pain score: the results of 5 studies (1879 patients) with combined data indicated that bisphosphonates therapy improved pain significantly assessed by WOMAC pain score (MD = 4.59; 95 % CI 2.83–6.34; P < 0.00001) compared with respective control group (Fig. 4).

**Fig. 5:**
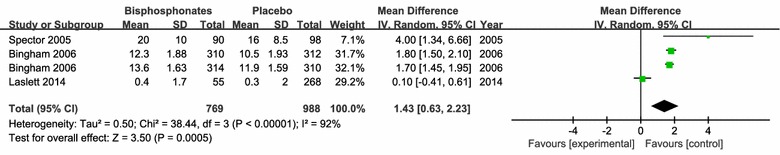
Change of WOMAC-Stiffness. The first Bingham et al. (2006) refers to the outcomes from 44 European centers (11 countries), the second Bingham refers to the outcomes from 42 centers in North America (US and Canada)

WOMAC stiffness score: the results of 3 studies (1757 patients) with combined data indicated that bisphosphonates therapy improved stiffness in affected joints significantly assessed by WOMAC stiffness score (MD = 1.43; 95 % CI 0.83–2.23; P = 0.0005) compared with respective control group (Fig. 5).

**Fig. 6:**
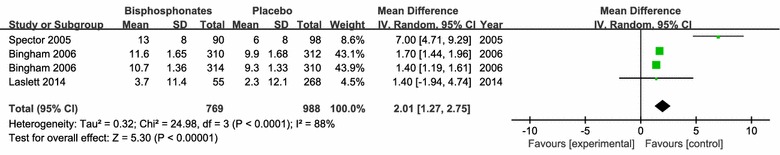
Change of WOMAC-Function. The first Bingham et al. (2006) refers to the outcomes from 44 European centers (11 countries), the second Bingham refers to the outcomes from 42 centers in North America (US and Canada)

WOMAC function score: the results of 3 studies (1757 patients) with combined data indicated that bisphosphonates therapy improved the function of affected joints significantly assessed by WOMAC function scores (MD = 2.01; 95 % CI 1.27–2.75; P < 0.00001) compared with respective control group (Fig. 6).

**Fig. 7:**
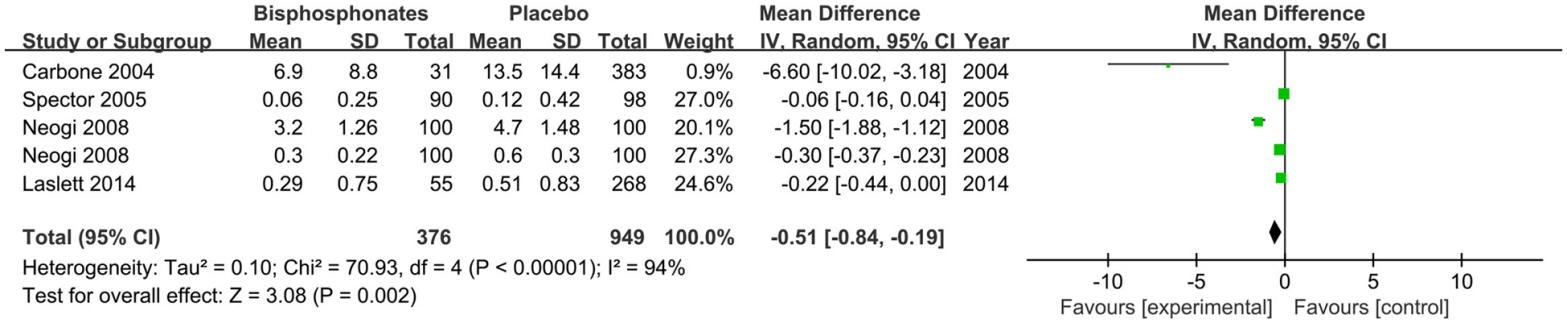
Change of Osteophyte Score. The first Neogi et al. (2008) refers to the outcomes of adjusted change in summary osteophyte score, the second Neogi et al. (2008) refers to the outcomes of adjusted change in summary disc-space narrowing score

Osteophyte score: the results of 4 studies (1125 patients) with combined data indicated that bisphosphonates therapy relieved osteophyte formation in affected joints significantly assessed by osteophyte score (MD = −0.51; 95 % CI −0.84 to −0.19; P = 0.002) compared with respective control intervention (Fig. 7).

### Secondary outcome

**Fig. 8:**
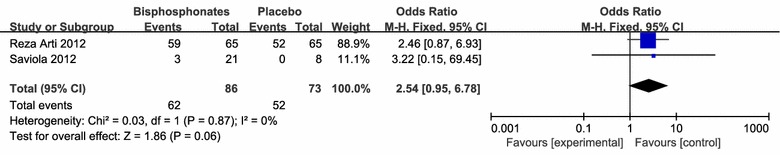
clinical improvement rate

**Fig. 9:**
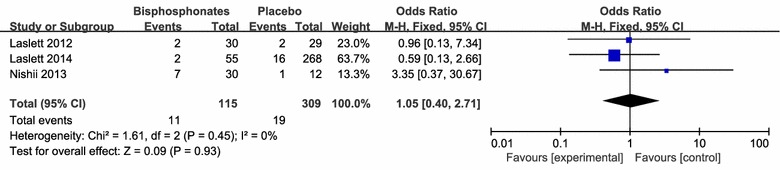
Joint replacement rate

**Fig. 10:**
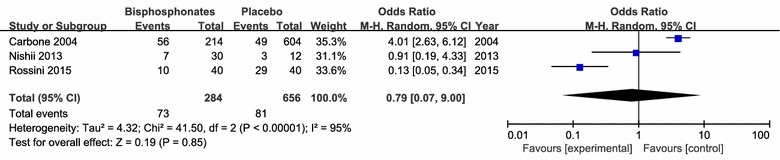
Patients required acetaminophen treatment

**Fig. 11:**
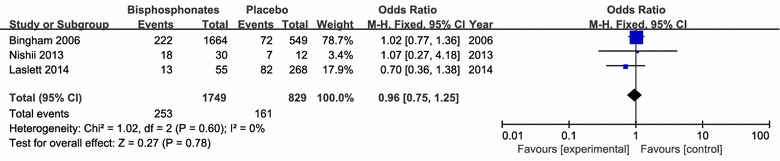
Osteoarthritis progression rate

There were no significant differences in osteoarthritis progression (3 studies including 2578 patients), required acetaminophen treatment (3 studies including 940 patients) and joint replacement (3 studies including 424 patients) between the application of bisphosphonates therapy and respective control intervention (Figs. 8, 9, 10, 11).

## Discussion

This meta-analysis demonstrated bisphosphonates therapy in OA had better effect on joint pain, stiffness and function, although it had no better effect on osteoarthritis progression, required acetaminophen treatment and joint replacement comparing to other medications. However, there was some heterogeneity across the included studies as for pain, stiffness and function scores, which may result from variation in the types or doses of bisphosphonates in different studies. When a sensitivity analysis was performed, the heterogeneity disappeared.

Current pharmacologic therapies for OA aim majorly to symptom control with analgesics, non-steroidal anti-inflammatory drugs (NSAIDs) and COX-2 inhibitors (Smith et al. 2016). NSAIDs, a most commonly used medication in symptomatic OA management, are found to be associated with gastrointestinal adverse reaction dose-related risks of renal toxicity and cardiovascular diseases (Brown 2013). Glucosamine has been suggested as a potential structure-modifying OA drug, but the data concerned are inconsistent (Krader 2014). Increased evidence suggests that high turnover metabolism derangement plays an important role in the initiation and progression of OA, which has resulted in an increased level of interest in drugs that affecting bone metabolism may slow the progression of OA (McGrory et al. 2016).

Based on 15 RCTs, this meta-analysis indicated that bisphosphonates therapy have better effect in relieving pain and accelerating functional recovery for OA. In our study, there were no significant differences on the number of required acetaminophen treatment and joint replacement between bisphosphonates therapy and other conventional medications. But the causes that patients received NSAID were various, and few researchers provided the details of clinical stage and pathological grade of their patients. Therefore, further studies are needed to provide more solid evidence.

## Quality of the evidence

The overall methodological quality was moderate, and most studies had at least one aspect of unclear or high risk of bias. Not all outcome assessors were blinded, which may produce performance or measurement biases. Selection bias may exist since only 3 studies used allocation concealment. As bisphosphonates are only be evaluated as “off label” therapy for OA, there was no corresponding standardized treatment guideline. Although we had performed sensitivity analysis, the effect of bisphosphonates may be underestimated.

## Conclusions

This meta-analysis showed that bisphosphonates therapy is effective for patients with OA in relieving pain and accelerating functional recovery. However, the conclusions are limited due to small sample sizes and methodological study quality, the different doses and treatment courses among studies. Further studies are needed to provide more solid evidence.
